# To Evaluate the Effect of Chronic Obstructive Pulmonary Disease on Retinal and Choroidal Thicknesses Measured by Optical Coherence Tomography

**DOI:** 10.1155/2019/7463815

**Published:** 2019-10-08

**Authors:** Sait Alim, Helin Deniz Demir, Ayşe Yilmaz, Selim Demir, Alper Güneş

**Affiliations:** ^1^Department of Ophthalmology, Gaziosmanpaşa University Faculty of Medicine, Tokat, Turkey; ^2^Department of Pulmonary Diseases, Hitit University School of Medicine, Çorum, Turkey

## Abstract

**Purpose:**

To evaluate the retinal and choroidal thicknesses in patients with chronic obstructive pulmonary disease using optical coherence tomography.

**Methods:**

The study included 26 patients with chronic obstructive pulmonary disease (COPD) and 26 age-matched healthy control groups. Detailed ocular examinations were performed on all participants. Cirrus EDI-OCT (enhanced depth imaging-optical coherence tomography) was used for choroidal thickness measurements with frame enhancement software. The subfoveal area was used for choroidal thickness measurements.

**Results:**

The patients with the chronic obstructive pulmonary disease had an average 239.13 ± 57.77 *μ*m subfoveal choroidal thickness, and the control group had an average 285.02 ± 25 *μ*m subfoveal choroidal thickness. The subfoveal choroidal thickness measurements revealed a statistically significant difference between patients and the control group (*p* < 0.05). There were no statistically significant differences between patients and control group regarding mean macular thickness, central macular thickness, and GCIPL (ganglion cell-inner plexiform layer) thickness. Also, there was no statistically significant difference between patients and control group regarding mean, superior, nasal, inferior, and temporal RNFL (retinal nerve fiber layer) thicknesses.

**Conclusion:**

Chronic hypoxemia seems to cause decreased choroidal thickness in patients with chronic obstructive pulmonary disease.

## 1. Introduction

Chronic obstructive pulmonary disease (COPD) is characterized by airflow limitation and persistent respiratory symptoms due to airway and/or alveolar abnormalities which are caused by exposure to gases and deleterious particles. The disease is preventable and treatable [1]. Cigarette smoking is the primary cause of the disease [2]. The prevalence of the COPD is estimated to be nearly 10% [3, 4]. Spirometry is required for diagnosis. Forced expiratory volume in one second (FEV1)/forced vital capacity (FVC) ≤0.7 and FEV1 ≥80 confirms the presence of airflow limitation that is not fully reversible which should be done after using a bronchodilator [5]. Although COPD is known primarily as lung disease, it can also produce significant systemic consequences because of smoking, increased systemic inflammation, tissue hypoxia-related sympathetic activity, procoagulant state, and arterial stiffness [3].

Retina and choroid is a complex microvascular system which can be affected by systemic diseases. Choroidal thickness in smoking and obstructive sleep apnea syndrome (OSAS) has already been studied and showed to be decreased in the central area as a result of chronic hypoxemia, vascular dysregulation, decreased nitric oxide, increased sympathetic activity, and systemic inflammation [6]. To the best of our knowledge, this is the first study which revealed statistically significant difference between patients and control group regarding subfoveal choroidal thickness (SCT) in COPD patients and spectral domain optical coherence tomography (SD-OCT); a noninvasive test was used to measure choroidal thickness which has been believed to be the predictor of the healthy choroid [7, 8].

## 2. Materials and Methods

Twenty-six patients with COPD and 26 age-sex-matched healthy participants as a control group were enrolled in this prospective case-control study. The study was conducted according to the guidelines of the Declaration of Helsinki, and patients provided their informed consent after the nature and purpose of the study were fully explained to them.

The study included the patients with COPD with duration of at least 10 years. The spirometric test was performed using Jaeger platform (Viays Healthcare GmbH, Aps-pro, Germany) to the patient and control groups. According to GOLD Guidelines (Global Initiative for Chronic Obstructive Lung Disease), COPD patients were established and classified [9].

Detailed ocular examinations were done for each participant. The patients and control group had no ocular disease except mild cataract. The best-corrected visual acuity (BCVA) of our patients and control group were logMAR 0.00. The axial length of the participants was 20–24 mm. Cycloplegic refractive errors greater than ±1.00 diopter (D) were excluded. By adding the sum of the sphere power with half of the cylinder power, the refractive error was calculated. For the spherical lens which would convert a case of the simple, compound or unequally mixed astigmatism into a case of equally mixed astigmatism used the term of spherical equivalent [10]. Also, control participants with cigarette smoking, exposure to the noxious gases, and working in a dusty area were excluded.

**Figure 1:**
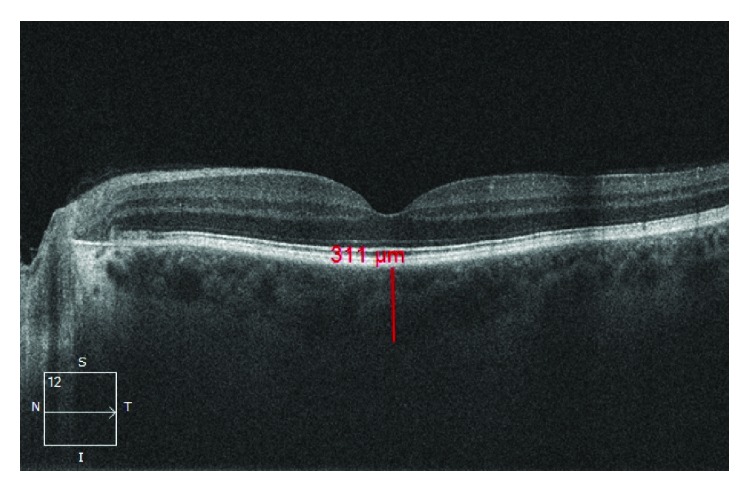
Subfoveal choroidal thickness of a control group.

**Figure 2:**
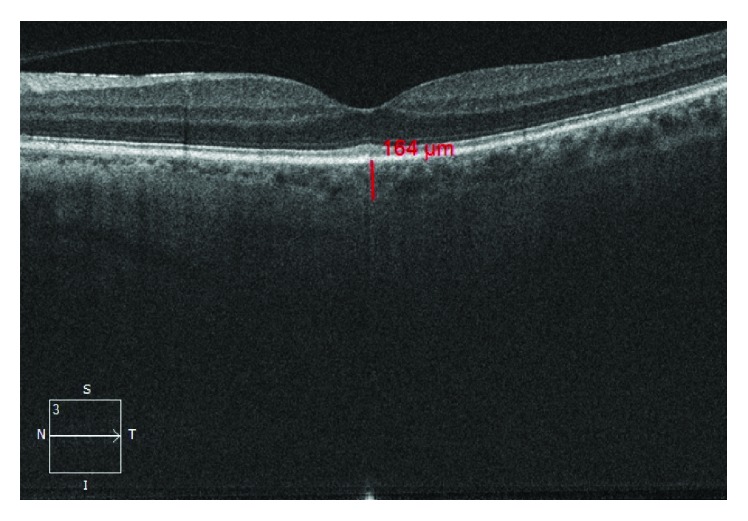
Subfoveal choroidal thickness of a patient group.

OCT measurements were performed by one experienced examiner. The patients and controls were analyzed with undilated pupils. SCT measurements were determined manually from the outer border of hyperreflective line coinciding with the retinal pigment epithelium to the inner surface of the sclera (Figures 1 and 2).

The choroidal thickness measurements were performed manually by two masked authors at different times (Dr. SA and Dr. HDD). The data evaluated by two observers were used in statistical analysis. Interobserver correlation was found.

Only the right eye of each study participant was assessed. The measured retinal parameters were as follows: central macular thickness, mean macular thickness, macular volume, peripapillary RNFL, and GCIPL thicknesses. Cirrus SD-OCT (Carl Zeiss Meditec, Dublin, CA) was used for scans of the macula (macular cube 512 × 128 × 1024 protocols) and the optic discs (optic disc cube 200 × 200 protocols). To analyze mean GCIPL thickness, the scan protocol of the macular cube (512 × 128 × 1024) was used. A signal power greater than 7 (Signal-to-noise ratio >7) was selected for the study. The axial length of the participants was measured with a biometer (EchoScan, US 4000, Nidek, Gamagori, Japan).

Four quadrants (superior, inferior, nasal, and temporal) of peripapillary RNFL thickness were measured, and averages of these four measurements were assessed.

### 2.1. Statistical Analysis

Quantitative data were obtained regarding the arithmetic mean and standard deviation. Qualitative data were found regarding frequency distribution tables. Independent samples *t* test or one-way ANOVA tests were used for variable comparisons between/among groups. A *p* value <0.05 was considered significant. Statistical analyses were performed using IBM SPSS Statistics 20.

## 3. Results

The spirometric and SCT measurements are demonstrated in Table 1. The average forced expiratory volume in 1 second (FEV1) was 2.90 ± 1.02 (lt) and 1.27 ± 0.44 (lt), respectively, in patients and control group (*p* < 0.001). The average forced vital capacity (FVC) was 3.49 ± 1.05 (lt) and 2.36 ± 0.61 (lt), respectively, in patients and control group (*p* < 0.001). The average FEV1/FVC ratio was 53.18 ± 10.55 and 78.90 ± 4.11, respectively, in patients and control group (*p* < 0.001).

The patients with the chronic obstructive pulmonary disease had an average 239.13 ± 57.77 *μ*m subfoveal choroidal thickness, and the control group had an average 285.02 ± 25.84 *μ*m subfoveal choroidal thickness.

There was a statistically significant difference between the patients and the control group regarding SCT (*p* < 0.001).

All our participants were male. The refractive error, BCVA, and axial length of the study group are demonstrated in Table 1.

There was no statistically significant difference between patients and control group regarding mean macular thickness and central macular thickness (*p* > 0.05). There was statistically significant difference between patients and control group regarding GCIPL thickness (*p*=0.026). It is demonstrated in Table 2.

As it is demonstrated in Table 3, there was no statistically significant difference between patients and control group regarding mean, superior, nasal, inferior, and temporal RNFL thicknesses (*p* > 0.05).

## 4. Discussion

The choroid is a highly vascular structure which has been accepted as a vascular bed of the eye and has a blood flow of the highest of any tissue in the body [11]. The metabolic and oxygen exchange of avascular area of the fovea is only supported by choroid, and the oxygen demands of the photoreceptors in the central macula are the highest. It has been mentioned that the choroidal thickness which can be evaluated by EDİ-OCT reveals the blood flow of the choroidal vasculature; and if the choroid is thin, there is less blood; conversely, if the choroid is thick, there is much blood in the choroidal vasculature [12, 13]. COPD can affect not only the lung system but also other organ systems of the body. Several studies have shown evidence of decreased vasodilation, increased atherosclerosis, arterial stiffness, risk of cardiovascular disease, and abnormalities in systemic vascular function in patients with COPD. The most common cardiovascular comorbidities seen in COPD are coronary artery disease, hypertension, atrial fibrillation, heart failure, stroke, and peripheral arterial disease, with a prevalence ranging from 28% to 70%. Endothelial dysfunction as a predictor of vascular dysfunction has been shown to be correlated with the severity of airflow obstruction in COPD patients. Disrupted flow-mediated dilation may show increased cardiovascular morbidity severely related to airway obstruction and systemic inflammation in COPD patients. We can conclude that endothelial dysfunction and systemic inflammation are both responsible for pathogenesis of atherosclerosis [14–20].

Retina and optic nerve have been shown to be effected in COPD patients by visual field and visual evoked potential (VEP) parameters [17]. COPD patients demonstrate raised sympathetic activity and circulating catecholamine and endothelin-1 (ET-1) and decreased nitric oxide (NO) levels which totally decrease the ocular perfusion pressure and choroidal blood flow and result in choroidal thinning [21, 22].

There have been some studies regarding choroidal thickness in COPD patients. A study by Ozcimen et al. [13] measured peripapillary choroidal thickness using OCT in patients with COPD. However, they did not find peripapillary choroidal thickness significantly different from the control group but found only inferior peripapillary choroidal thickness in patients with COPD to be thinner than the other sectors of the choroid and the control group. They concluded that choroidal thinning might be due to vascular endothelial dysfunction and hypoxia, making the choroid more sensitive to retinal and choroidal diseases. Another study by Gok et al. [23] found no significant difference between COPD patients and the control group regarding choroidal thickness. They also found no significant difference in macular choroidal thickness when subgroup analyses were assessed as mild and severe COPD patients and the control group as well. They found GCIPL thinner in COPD patients, and they emphasized that GCIPL may be affected more in severe COPD patients.

We found GCIPL thinner in COPD patients, and it showed statistically significant difference between patients and control group. RNFL thickness revealed no statistically significant difference between patients and control group. We can speculate about that, as in glaucoma patients, maybe GCIPL thickness gets affected earlier than RNFL thickness in COPD patients. In glaucoma, decreased GCIPL thickness may be an early detector of ganglion cell loss which comes before axonal loss [24, 25].

We also found a statistically significant difference in COPD patients compared with the control group regarding SCT. According to our opinion, the difference might be due to that the patients in our study had severe COPD diseases different from the previous studies which had mild and severe COPD cases [13, 23].

Our present study has some limitations; firstly, the study sample size was relatively small. Secondly; the patients and control groups were composed of only male participants. Further studies are needed to evaluate the choroidal thickness in COPD patients with all disease stages and with large sample size.

In summary, conversely to the previous studies, our present study revealed a statistically significant difference between patients with COPD and control group regarding SCT. We think that SD-OCT is an important tool for monitoring COPD patients. Furthermore, SCT can be used for disease progression in COPD patients.

## Figures and Tables

**Table 1:** The age, refractive error, best-corrected visual acuity, axial length, and spirometric measurements of the study group.

	Control		Patient		*p*
	*N*	Mean ± SD	*N*	Mean ± SD	
Age (years)	26	58.19 ± 8.94	26	61.69 ± 7.2	0.126
Refractive error (D)	26	−0.68 ± 0.28	26	−0.54 ± 0.80	0.405
BCVA (logMAR)	26	0.00	26	0.00	1.000
Axial length (mm)	26	23 ± 2.25	26	22 ± 2.23	0.114
FEV1 (lt)	26	2.90 ± 1.02	26	1.27 ± 0.44	0.001
FVC (lt)	26	3.49 ± 1.05	26	2.36 ± 0.61	0.001
FEV1/FVC ratio	26	78.90 ± 4.11	26	53.18 ± 10.55	0.001

*p*: independent samples *t* test was used; FEV1: forced expiratory volume in 1 second; FVC: forced vital capacity; D: diopter; BCVA: best-corrected visual acuity.

**Table 2:** The subfoveal choroidal thickness, mean macular thickness, central macular thickness, and GCIPL thickness measurements.

	Control		Patient		*p*
	*N*	Mean ± SD	*N*	Mean ± SD	
Subfoveal choroidal Thickness (*μ*m)	26	285.02 ± 25.84	26	239.13 ± 57.77	0.001
Mean macular thicknes (*μ*m)	26	282.92 ± 13.91	26	275.35 ± 15.44	0.069
Central macular thickness (*μ*m)	26	255.85 ± 20.31	26	251.27 ± 22.43	0.444
GCIPL (*μ*m)	26	82.92 ± 7.36	26	76.77 ± 11.54	0.026

*p*: independent samples *t* test was used. GCILP: ganglion cell-inner plexiform layer.

**Table 3:** The mean, superior, nasal, inferior, and temporal RNFL thicknesses.

	Control		Patient		*p*
	*N*	Mean ± SD	*N*	Mean ± SD	
Mean RNFL (*μ*m)	26	92.62 ± 9.29	26	90.52 ± 10.8	0.479
Superior (*μ*m)	26	111.58 ± 15.23	26	109.67 ± 16.75	0.685
Nasal (*μ*m)	26	71.88 ± 7.06	26	69.81 ± 13.79	0.508
Inferior (*μ*m)	26	124.92 ± 14.03	26	119.62 ± 18.16	0.264
Temporal (*μ*m)	26	62.35 ± 13.81	26	62.71 ± 12.28	0.924

*p*: independent samples *t* test was used; *p*_2_: Hotelling's *T*^2^ test was used; RNFL: retinal nerve fiber layer.

## Data Availability

The data used to support the findings of this study are included within the article.
